# Risk of hearing loss in patients with fibromyalgia: A nationwide population-based retrospective cohort study

**DOI:** 10.1371/journal.pone.0238502

**Published:** 2020-09-03

**Authors:** Thi Phuong Le, Ya-Ling Tzeng, Chih-Hsin Muo, Hua Ting, Fung-Chang Sung, Shin-Da Lee, Yu-Kuei Teng

**Affiliations:** 1 Nursing Department, Duy Tan University, Da Nang, Vietnam; 2 School of Nursing and Graduate Institute of Nursing, China Medical University, Taichung, Taiwan; 3 Department of Nursing, China Medical University Hospital, Taichung, Taiwan; 4 Management Office for Health Data, China Medical University Hospital, Taichung, Taiwan; 5 Institute of Medicine, Chung-Shan Medical University, Taichung, Taiwan; 6 Sleep Medicine Center, Chung Shan Medical University Hospital, Taichung, Taiwan; 7 Department of Physical Medicine and Rehabilitation, Chung Shan Medical University Hospital, Taichung, Taiwan; 8 Department of Public Health, China Medical University, Taichung, Taiwan; 9 Department of Physical Therapy, Graduate Institute of Rehabilitation Science, China Medical University, Taichung, Taiwan; 10 Department of Healthcare Administration, Asia University, Taichung, Taiwan; University of Valencia, SPAIN

## Abstract

**Objectives:**

Our objectives are to examine whether patients with fibromyalgia (FM) present an increased risk of hearing loss (HL) compared with those without FM and to explore the relationship between comorbidities/drugs and development of HL in patients with FM. Furthermore, we investigated the incidence rate of different types of HL and the joint effects for HL with FM and comorbidities.

**Methods:**

This population-based retrospective cohort study included patients with new-onset FM from 2000 to 2002 (the FM group) and age- and sex-matched randomized patients without FM (the non-FM group) from Taiwan’s National Health Insurance Research Database. Patients were followed up from baseline (3 months after FM diagnosis) until death, withdrawal, HL development, or December 31, 2013. The primary outcome was the risk of HL, which was assessed using Cox proportional-hazards analysis.

**Results:**

The overall HL risk in the FM group was 1.46-fold (95% confidence interval [CI]: 1.38–1.55) higher than that in the non-FM group after adjustment for sex, age, and comorbidities (p < 0.0001). Patients with FM had significantly greater sensorineural HL (adjusted hazard ratio = 1.46, 95% CI: 1.37–1.56) than those without FM. Patients with FM having comorbidities of diabetes, hyperlipidemia, depression, and Meniere’s disease had a higher risk of HL than those without FM.

**Conclusion:**

Our findings support the notion that FM influences HL and is in line with the hypothesis that the FM mechanism is related to a central nervous system abnormality in sensory processing. Health care professionals should provide appropriate screening for the risk of HL and prevention and counseling methods for patients with FM.

## Introduction

Fibromyalgia (FM) is a musculoskeletal condition characterized by chronic widespread pain and specific tender points [1]. Its prevalence in the general population is 0.2%–4.7% and increases from the middle age; it is more common in women than in men [2, 3]. Patients with FM often have nonspecific symptoms and are at increased risk of many comorbidities and disorders [4]. Recently, a mechanism underlying FM etiopathology was hypothesized, which led to evidence suggesting that people with FM also may be at risk of hearing loss (HL) [5, 6]. The etiopathogenesis of FM remains clear, but it appears to be complex and multifactorial, involving familial, genetic, environmental, endocrine, and neurological system (both central and peripheral) factors. Dysregulation of the central nervous system (CNS) and peripheral nervous system affects FM development, which might be associated with the activation of nociceptive pathways, further contributing to central sensitization. Central sensitization involves CNS pain amplification and neuroendocrine dysfunctions, including decreased levels of serotonin (which inhibits pain signals) and increased levels of substance P (which propagates pain signals) [7, 8], leading to abnormal pain processing [7]. Thus, FM seems to be closely linked to central sensitivity syndromes, and patients with FM seem to have a nervous system that is overly sensitive to all stimuli [5]. Furthermore, patients with FM might also have brainstem dysfunction, which may cause abnormalities in processing auditory stimuli [5, 6]. Taken together, these findings imply that patients with FM have hypersensitivity toward not only pain but also sensory processing such as hearing.

HL is a leading cause of disability in adults [9] and causes serious communication and psychosocial problems and reduced quality of life. HL may be the result of damage to any part of the peripheral and central auditory systems [9]. Peripheral HL may be conductive (outer or middle ear impairment), sensorineural (cochlear or spiral ganglion dysfunction), or mixed. Neural HL is due to loss or dysfunction of spiral ganglion neurons or of more proximal auditory structures. The etiology of HL includes degenerative processes associated with aging, genetic mutations, exposure to noise, drug-induced adverse effects, and chronic diseases [9]. Studies on HL in people with FM have reported conflicting results [10–15], and long-term population-based cohort studies on the link between HL risk in patients with FM are lacking. Additionally, comorbidities and use of drugs in patients may also relate to HL development [16–22]. However, no study has examined the risk of HL in patients with FM stratified by these comorbidities, HL-inducing drugs, and drugs commonly used in patients with FM, such as antidepressants and pain-relieving drugs. To the best of our knowledge, retrospective cohort studies investigating whether FM influences HL and whether an association exists between comorbidities, drugs, and the risk of HL in patients with FM are scarce.

In this study, we used the Taiwan National Health Insurance Research Database (NHIRD), which has a large sample size. The study hypothesis was that patients with FM had a higher risk of HL than those without FM, after adjustment for sex, age, and comorbidities. More specifically, this study investigated whether people with FM have a significantly higher risk of HL than people without FM and whether the common comorbidities and drugs in patients with FM are associated with a risk of HL.

## Methods

### Data source

In the present population-based cohort study, we used the Longitudinal Health Insurance Database (LHID) 2000, which was retrieved from the NHIRD, Taiwan. Taiwan's National Health Insurance (NHI) system is a single-payer mandatory program, which covers >99% of Taiwan's population. The LHID 2000 contains the medical reimbursement claims data of 1 million people randomly sampled from the entire population registered in 2000 and is thus representative of the whole population. The disease diagnoses were encoded in accordance with the International Classification of Diseases, Ninth Revision, Clinical Modification (ICD-9-CM). For protecting personal privacy, patients' personal information was encrypted in the released database. Therefore, patient consent is not required to access the NHIRD. This study was approved by the Institutional Review Board of China Medical University (CMUH104-REC2-115[CR-2]). The NHIRD was used for data analysis with the approval of the NHI administration.

### Criteria for selecting patients

**Fig 1:**
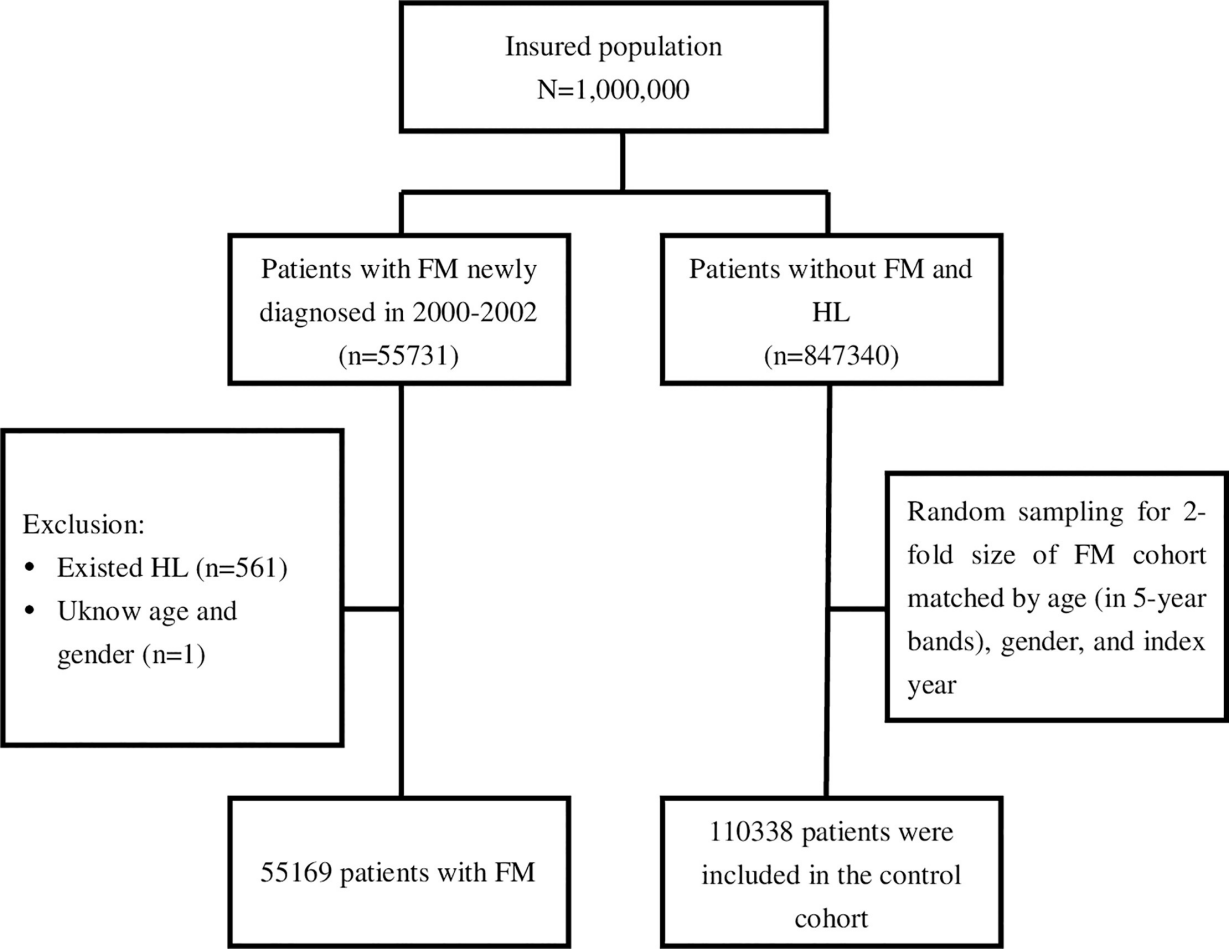
Flowchart for establishing study cohorts with and without fibromyalgia.

Fig 1 illustrates the process of identifying patients for this study. We used the diagnostic codes of the ICD-9-codes to identify patients with HL (ICD-9- codes 389, 389.1, and 389.2) and FM (ICD-9-codes 729.0 and 729.1). The ICD-9-codes of subtypes of sensorineural HL include bilateral sensory HL (ICD-9-code 389.11), bilateral neural HL (ICD-9-code 389.12), unilateral neural HL (389.13), and central HL (389.14). We identified an FM cohort and a non-FM cohort from the LHID2000. Patients who were newly diagnosed as having FM for at least 3 months (defined as baseline) between 2000 and 2002 (n = 55,169) were included as the study group (FM group). The date of FM diagnosis was defined as the index date. We excluded those who had HL at baseline and those for whom information on sex or age was missing. Each FM patient in this study was randomly frequency matched with two patients without FM and HL according to age (in 5-year bands), sex, and index year from the LHID2000 to obtain the non-FM group (n = 110,338). Participants were tracked from baseline until development of HL, loss to follow-up, withdrawal from NHI, or December 31, 2013 to examine the risk of HL in patients with FM compared with controls.

### Variables of comorbidity and drugs

Baseline comorbidities and diseases considered as potential covariates in the inferring of association between FM and HL included the following: diabetes (ICD-9-code 250), hypertension (ICD-9-codes 401–405), hyperlipidemia (ICD-9-code 272), depression (ICD-9-codes 296.2, 296.3, 300.4, and 311), anxiety (ICD-9-codes 300.01, 300.02, 300.2, 300.3, 308, and 309.81), Meniere's disease (ICD-9-code 386), insomnia (ICD-9-codes 780.52, 307.40, 307.41, 307.42, 307.43, and 307.44), and autoimmune diseases, including psoriasis (ICD-9-code 696), systemic lupus erythematosus (ICD-9-code 710.0), systemic sclerosis (ICD-9-code 710.1), Sjogren syndrome (ICD-9-code 710.2), dermatomyositis (ICD-9-code 710.3), polymyositis (ICD-9-code 710.4), and vasculitis (ICD-9-codes 446.0, 446.2, 446.4, 446.5, 443.1, 446.7, 446.1, and 136.1).

Pain-relieving drugs comprises dopamine-receptor agonists, serotonin 5-HT3 receptor antagonists, anti-epileptics, and opioid drugs. HL-inducing drugs include antimalarial drugs, aminoglycoside antibiotic drugs, tetracycline antibiotic drugs, other antibiotic drugs, macrolide antibiotic drugs, phosphodiesterase type 5 inhibitors, aspirin, diuretic drugs, and antitumor drugs/chemotherapeutic agents.

### Statistics

SAS software version 9.1 (SAS Institute, Cary, NC, USA) was used to calculate descriptive and inferential statistics. χ^2^ test for categorical data and Student's *t* tests for continuous data were used to compare intergroup differences in terms of sex, age, and comorbidities. The incidence rates of HL (per 1000 person-years) and the FM cohort to non-FM cohort hazard ratio (HR) of HL with 95% confidence interval (CI) were calculated using the Cox proportional-hazards analysis. Survival was analyzed with the Kaplan–Meier method, and the cumulative incidence of HL was compared between the FM and non-FM cohorts by using the log-rank test. The statistical significance level was set as P < 0.05.

## Results

In this retrospective cohort study, we selected 55,169 patients with FM for the FM group and 110,338 matched patients in sex and age for the non-FM group (1:2 match ratio). In all, 59.4% of the participants were female and 40.6% were male, and the predominant age group was 40–59 years (40.6%; Table 1). The FM group had a higher percentage of comorbidities than the non-FM group, including diabetes, hypertension, hyperlipidemia, depression, anxiety, Meniere's disease, insomnia, and autoimmune diseases. Patients with FM had a significantly higher prevalence of using antidepressants, pain-relieving drugs, and HL-inducing drugs.

**Table 1:** Distribution of sex, age, and comorbidity between patients with and without fibromyalgia.

	Fibromyalgia (N = 55169)	Comparison (N = 110338)	
Variable	N(%)	N(%)	P-value
Sex					0.99
Female	32756(59.4)	65512(59.4)	
Male	22413(40.6)	44826(40.6)	
Age, year			0.99
<40	20557(37.3)	41114(37.3)	
40–59	22419(40.6)	44838(40.6)	
60+	12193(22.1)	24386(22.1)	
Mean (SD)	45.9(16.9)	45.8(17.0)	0.56
Comorbidity			
Diabetes	6476(11.7)	10062(9.12)	<0.0001
Hypertension	14681(26.6)	22656(20.5)	<0.0001
Hyperlipidemia	9928(18.0)	12612(11.4)	<0.0001
Depression	2136(3.87)	2030(1.84)	<0.0001
Anxiety	626(1.13)	580(0.53)	<0.0001
Meniere’s disease	4180(7.58)	3937(3.57)	<0.0001
Insomnia	3961(7.18)	3533(3.20)	<0.0001
Autoimmune diseases ^a^	271(0.49)	421 (0.38)	0.001
Medicine ^b^			
Anti-depression	4210(7.63)	4099(3.71)	<0.0001
Pain-relieving drug ^c^	3597(6.52)	3593(3.26)	<0.0001
Hearing loss-inducing drug ^d^	26509(48.1)	35985 (32.6)	<0.0001

^a^ Autoimmune disease: psoriasis, systemic lupus erythematosus, systemic sclerosis, Sjogren syndrome, dermatomyositis, polymyositis, and vasculitis.

^b^ Patients who underwent medical treatment within 1 year before the index date.

^c^ Pain-relieving drugs: dopamine-receptor agonists, serotonin 5-HT3 receptor antagonists, antiepileptics, and opioid analgesics.

^d^ Hearing loss–inducing drugs: antimalarial drugs, aminoglycoside antibiotic drugs, tetracycline antibiotic drugs, other antibiotic drugs, macrolide antibiotic drugs, phosphodiesterase type 5 inhibitors, aspirin, diuretic drugs, and antitumor drugs/chemotherapeutic agents.

**Fig 2:**
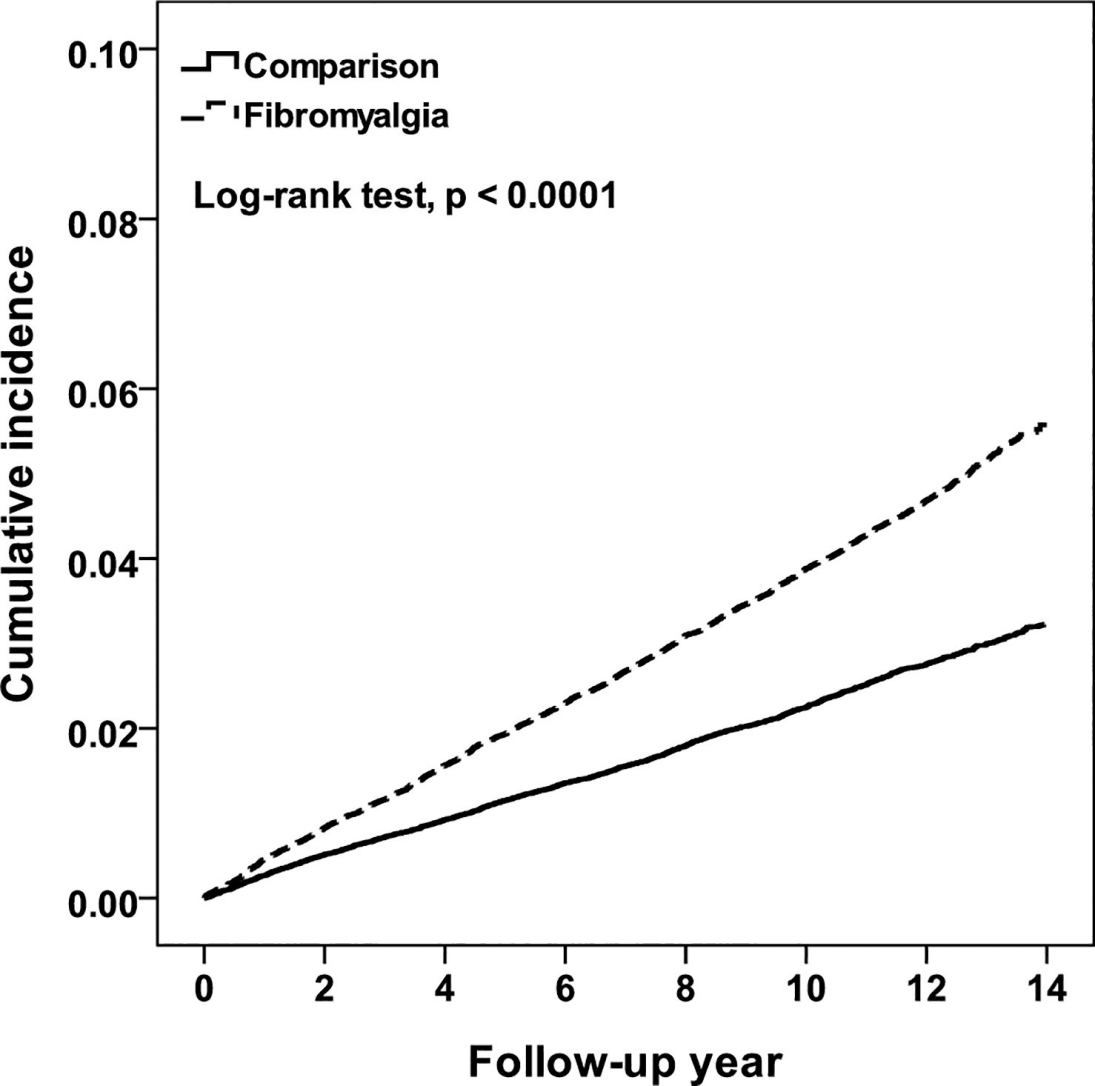
Cumulative incidence for hearing loss between patients with and without fibromyalgia.

Fig 2 shows that the cumulative HL incidence was higher in the FM group (4.03 per 1000 person-years) than in the non-FM group (2.33 per 1000 person-years; log-rank P < 0.0001) at the end of the follow-up period (Table 2). The overall HL risk in the FM group was 1.46-fold (95% CI: 1.38–1.55) higher than that in the non-FM group after adjustment for sex, age, and comorbidities (p < 0.0001). In general, the incidence of HL was higher in men than in women. The risk of HL in men was 1.45-fold (95% CI: 1.37–1.54, p < 0.0001) higher than that in women after adjustment for sex, age, and comorbidities. Compared with those aged <40 years, the risk of HL was higher in older patients and increased with age. Stratified by comorbidities, the crude HRs indicated that patients with comorbidities had a 1.68–3.53-fold increased risk of HL than patients without comorbidities. After adjustment for FM, sex, age, and drugs, the risk of HL in patients with comorbidities such as diabetes, hyperlipidemia, depression, and Meniere's disease was significantly higher than that in those without these comorbidities. Stratified by drugs, people taking antidepressants and HL-inducing drugs had higher risks of HL than those who were not taking these drugs.

**Table 2:** Incidence and risk of hearing loss and associated risk factors.

						HR (95% CI)	
Variable	Hearing loss no.	Person-years	Rate	Crude	p	Adjusted	P-value
Fibromyalgia							
No	2186	9374718	2.33	Ref.		Ref.	
Yes	2590	6442239	4.03	1.72 (1.63–1.82)	<0.0001	1.46 (1.38–1.55)	<0.0001
Sex							
Female	2454	929358	2.64	Ref.		Ref.	
Male	2322	650299	3.57	1.35 (1.28–1.43)	<0.0001	1.45 (1.37–1.54)	<0.0001
Age							
< 40	737	636945	1.16	Ref.		Ref.	
40–59	1870	648135	2.89	2.50 (2.30–2.72)	<0.0001	2.24 (2.06–2.45)	<0.0001
60+	2169	294576	7.36	6.46 (5.94–7.02)	<0.0001	4.71 (4.28–5.17)	<0.0001
Comorbidity							
Diabetes							
No	3890	1442987	2.70	Ref.		Ref.	
Yes	886	136669	6.48	2.42 (2.25–2.60)	<0.0001	1.11 (1.03–1.21)	0.01
Hypertension							
No	2809	1263284	2.22	Ref.		Ref.	
Yes	1967	316372	6.22	2.81 (2.66–2.98)	<0.0001	1.06 (0.99–1.14)	0.11
Hyperlipidemia							
No	3550	1379315	2.57	Ref.		Ref.	
Yes	1226	200341	6.12	2.38 (2.23–2.54)	<0.0001	1.21 (1.13–1.31)	<0.0001
Depression							
No	4536	1542623	2.94	Ref.		Ref.	
Yes	240	37034	6.48	2.21 (1.94–2.52)	<0.0001	1.18 (1.02–1.37)	0.03
Anxiety							
No	4721	1568726	3.01	Ref.		Ref.	
Yes	55	10930	5.03	1.68 (1.28–2.18)	0.0001	0.96 (0.73–1.25)	0.74
Meniere’s disease							
No	4108	1509853	2.72	Ref.		Ref.	
Yes	668	69804	9.57	3.53 (3.25–3.83)	<0.0001	1.84 (1.68–2.00)	<0.0001
Insomnia							
No	4357	1514251	2.88	Ref.		Ref.	
Yes	419	65406	6.38	2.22 (2.01–2.46)	<0.0001	1.11 (0.97–1.23)	0.06
Autoimmune diseases ^a^							
No	4754	1573486	3.02	Ref.			
Yes	22	6171	3.57	1.18 (0.78–1.80)	0.43		
Medicine							
Anti-depression							
No	4295	1507950	2.85	Ref.		Ref.	
Yes	481	71706	6.71	2.36 (2.14–2.59)	<0.0001	1.29 (1.16–1.43)	<0.0001
Pain-relieving drug ^b^							
No	4425	1517824	2.92	Ref.		Ref.	
Yes	351	61832	5.68	1.95 (1.75–2.17)	<0.0001	1.10 (0.98–1.23)	0.11
Hearing loss-inducing drug ^c^							
No	2610	999496	2.61	Ref.		Ref.	
Yes	2166	580160	3.73	1.43 (1.35–1.51)	<0.0001	1.11 (1.05–1.18)	0.0005

^a^ Autoimmune disease: psoriasis, systemic lupus erythematosus, systemic sclerosis, Sjogren syndrome, dermatomyositis, polymyositis, and vasculitis.

^b^ Pain-relieving drugs: dopamine-receptor agonists, serotonin 5-HT3 receptor antagonists, antiepileptics, and opioid analgesics.

^c^ Hearing loss–inducing drugs: antimalarial drugs, aminoglycosides antibiotic drugs, tetracycline antibiotic drugs, other antibiotic drugs, macrolide antibiotic drugs, phosphodiesterase type 5 inhibitors, aspirin, diuretic drugs, and antitumor drugs/chemotherapeutic agents.

Results in Table 3 indicated that patients with FM had a significantly increased risk of HL than those without FM with overall (aHR = 1.45, CI: 1.37–1.53, p < 0.0001), conductive (aHR = 1.34, CI: 1.01–1.78, p = 0.044), sensorineural (aHR = 1.46, CI: 1.37–1.56, p < 0.0001), and mixed HL (aHR = 1.56, CI: 1.20–2.02, p = 0.0008) after adjustment for age, sex, comorbidities, and drugs. Among patients with FM with sensorineural HL, the most common type is sensory HL, bilateral (aHR = 1.32, CI: 1.02–1.71, p = 0.03). Patients with FM had a higher risk of sensorineural HL than of conductive HL compared with the non-FM group.

**Table 3:** Incidence and risk of different types of hearing loss.

	Fibromyalgia			Comparison				
Outcome	Hearing loss no.	Person-years	Rate	Hearing loss no.	Person-years	Rate	Adjusted HR (95% CI)	p
Overall	2590	6442239	4.03	2186	9374718	2.33	1.45 (1.37–1.53)	<0.0001
Conductive	108	642236	0.17	102	937418	0.11	1.34 (1.01–1.78)	0.044
Sensorineural	2339	642239	3.64	1970	937418	2.10	1.46 (1.37–1.56)	<0.0001
Sensory hearing loss, bilateral	126	642239	0.20	129	937418	0.14	1.32 (1.02–1.71)	0.03
Neural hearing loss, bilateral	34	642239	0.05	44	937418	0.05	0.84 (0.52–1.34)	0.46
Neural hearing loss, unilateral	0	642239	0.00	0	937418	0.00	NA	
Central hearing loss	5	642239	0.01	5	937418	0.01	1.16 (0.32–4.13)	0.82
Other	2174	642239	3.39	1792	937418	1.91	1.49 (1.39–1.59)	<0.0001
Mixed	143	642239	0.22	114	937418	0.12	1.56 (1.20–2.02)	0.0008

Adjusted for age, sex, diabetes, hypertension, hyperlipidemia, depression, anxiety, Meniere’s disease, insomnia, antidepressants, pain-relieving drugs, and hearing loss–inducing drugs.

The adjusted HRs in women and men were similar in both groups (aHR = 1.50, 95% CI: 1. 38–1.63 [women] vs. aHR = 1.41, 95% CI: 1. 29–1.54 [men]) after controlling for sex, age, comorbidity, and drugs in Table 4. Stratified by age, the risk of HL in the patients with FM was significantly higher than that in the patients without FM aged <40 (aHR = 1.73, 95% CI: 1.48–2.01, p < 0.0001), 40–59 (aHR = 1.44, 95% CI: 1.31–1.58, p < 0.0001), and ≥60 (aHR = 1.35, 95% CI: 1.23–1.47, p < 0.0001). Patients with FM <40 years old had a higher risk of HL than those without FM in the other age groups.

**Table 4:** Incidence and risk of hearing loss stratified by sex, age group, comorbidity, and drug.

	Fibromyalgia			Comparison				
Variable	Hearing loss no.	Person-years	Rate	Hearing loss no.	Person-years	Rate	Adjusted HR (95% CI)	p
Sex								
Female	1382	387094	3.57	1072	542263	1.98	1.50 (1.38–1.63)	<0.0001
Male	1208	255144	4.73	1114	395154	2.82	1.41 (1.29–1.54)	<0.0001
Age, year								
< 40	414	250440	1.65	323	386505	0.84	1.73 (1.48–2.01)	<0.0001
40–59	1046	268150	3.90	824	379985	2.17	1.44 (1.31–1.58)	<0.0001
60+	1130	123648	9.14	1039	170928	6.08	1.35 (1.23–1.47)	<0.0001
Comorbidity								
Diabetes								
No	2074	573223	3.62	1816	869764	2.09	1.48 (1.38–1.58)	<0.0001
Yes	516	59015	7.48	370	67654	5.47	1.35 (1.18–1.56)	<0.0001
Hypertension								
No	1457	484432	3.01	1352	778852	1.74	1.54 (1.42–1.66)	<0.0001
Yes	1133	157806	7.18	834	158566	5.26	1.31 (1.20–1.44)	<0.0001
Hyperlipidemia								
No	1835	530273	3.46	1715	849042	2.2	1.53 (1.43–1.64)	<0.0001
Yes	755	111966	6.74	471	88375	5.33	1.22 (1.08–1.38)	0.001
Depression								
No	2412	618652	3.90	2124	923970	2.30	1.45 (1.36–1.54)	<0.0001
Yes	178	23586	7.55	62	13447	4.61	1.68 (1.24–2.26)	0.0007
Anxiety								
No	2549	635160	4.01	2172	933566	2.33	1.46 (1.37–1.55)	<0.0001
Yes	41	7079	5.79	14	3852	3.63	1.70 (0.90–3.21)	0.10
Meniere’s disease								
No	2119	597346	3.55	1989	912507	2.18	1.45 (1.36–1.55)	<0.0001
Yes	471	44892	10.49	197	24911	7.91	1.41 (1.18–1.67)	0.0001
Insomnia								
No	2296	599029	3.83	2063	915222	2.25	1.47 (1.38–1.57)	<0.0001
Yes	294	43210	6.80	123.	22196	5.54	1.23 (0.99–1.53)	0.07
Autoimmune diseases ^a^								
No	2576	639353	4.03	2178	934133	2.33	1.46 (1.37–1.55)	<0.00001
Yes	14	2885	4.85	8	3285	2.44	1.80 (0.73–4.40)	0.20
Medicine								
Anti-depression								
No	2237	596237	3.75	2058	911714	2.26	1.44 (1.35–1.53)	<0.0001
Yes	353	46002	7.67	128	25704	4.98	1.62 (1.31–1.99)	<0.0001
Pain-relieving drug ^b^								
No	2343	603160	3.88	2082	914664	2.28	1.47 (1.38–1.56)	<0.0001
Yes	247	39079	6.32	104	22753	4.57	1.31 (1.04–1.66)	0.02
Hearing loss-inducing drug ^c^								
No	1233	335870	3.67	1377	663626	2.07	1.56 (1.44–1.69)	<0.0001
Yes	1357	306369	4.43	809	273791	2.95	1.33 (1.21–1.45)	<0.0001

Manually adjusted for age, sex, diabetes, hypertension, hyperlipidemia, depression, anxiety, Meniere’s disease, insomnia, pain-releasing drug, and hearing loss-inducing drug.

^a^ Autoimmune disease: psoriasis, systemic lupus erythematosus, systemic sclerosis, Sjogren syndrome, dermatomyositis, polymyositis and vasculitis.

^b^ Pain-relieving drugs: dopamine-receptor agonists, serotonin 5-HT3 receptor antagonists, antiepileptics, and opioid analgesics.

^c^ Hearing loss–inducing drugs: antimalarial drugs, aminoglycosides antibiotic drugs, tetracycline antibiotic drugs, other antibiotic drugs, macrolide antibiotic drugs, phosphodiesterase type 5 inhibitors, aspirin, diuretic drugs, and antitumor drugs/chemotherapeutic agents.

The incidence rate of HL was higher in patients with comorbidities (diabetes, hypertension, hyperlipidemia, depression, and Meniere’s disease) than in those without comorbidities in both the FM and non-FM groups. Nevertheless, after adjustment for sex, age, comorbidities, and drugs, patients with FM had significantly higher risks of HL than those without FM regardless of whether they had these comorbidities (all p < 0.05). Patients taking antidepressants, pain-relieving drugs, and HL-inducing drugs exhibited increased HL incidence rates in both FM and non-FM groups. However, after adjustment for sex, age, comorbidities, and drugs, patients with FM still had a higher risk of HL than those in the non-FM group irrespective of whether they used these drugs after adjustment for sex, age, comorbidity, and drugs (all p < 0.05).

Regarding the joint effects of various comorbidities on the risk of HL, Table 5 shows the results of regression models adjusted for age, sex, pain-relieving drugs, and HL-inducing drugs. Compared with patients without any comorbidity (reference group), the independent effect of FM on the risk of HL was statistically significant (aHR = 1.63, 95% CI: 1.49–1.78, p < 0.0001). Patients with Meniere's disease were at the highest risk of HL among patients with only one comorbidity (aHR = 2.80, 95% CI: 2.09–3.76, p < 0.0001). Among patients with FM with one comorbidity, those with Meniere’s disease exhibited the highest risk (aHR = 4.01, 95% CI: 3.27–4.94), followed by those with hyperlipidemia, diabetes, insomnia, hypertension, and depression (S1 Appendix). Patients with FM with any five comorbidities exhibited the highest risk of HL (aHR = 3.71, 95% CI: 2.64–5.23, p < 0.0001; Table 5).

**Table 5:** Joint effects of comorbidities on hearing loss.

Variables	N	Hearing loss no.	Person-years	Rate	Adjusted HR (95% CI)	p
None	77688	1062	707150	1.50	Ref.	
Only Fibromyalgia	32017	915	385773	2.37	1.63 (1.49–1.78)	<0.0001
Only Diabetes	1759	42	13053	3.22	1.28 (0.94–1.74)	0.12
Only Hypertension	10029	300	74430	4.03	1.28 (1.12–1.46)	0.0003
Only Hyperlipidemia	2994	74	22379	3.31	1.53 (1.21–1.94)	0.0004
Only Depression	636	14	4655	3.01	1.59 (0.94–2.70)	0.09
Only Meniere’s disease	1151	47	8028	5.85	2.80 (2.09–3.76)	<0.0001
Only Insomnia	1032	24	7113	3.37	1.75 (1.17–2.62)	0.007
Fibromyalgia with any one comorbidity	11577	671	131845	5.09	2.03 (1.83–2.24)	<0.0001
Fibromyalgia with any two comorbidities	6607	536	72209	7.42	2.49 (2.23–2.79)	<0.0001
Fibromyalgia with any three comorbidities	3616	309	38551	8.02	2.43 (2.13–2.79)	<0.0001
Fibromyalgia with any four comorbidities	1072	121	11073	10.93	3.03 (2.49–3.69)	<0.0001
Fibromyalgia with any five comorbidities	245	35	2459	14.23	3.71 (2.64–5.23)	<0.0001
Fibromyalgia with any all comorbidities	35	3	329	9.11	2.13 (0.68–6.63)	0.19
Only comorbidity more than one	15049	623	100608	6.19	1.93 (1.74–2.15)	<0.0001

Adjusted for age, sex, antidepressants, pain-relieving drugs, and hearing loss–inducing drugs.

Comorbidities included diabetes, hypertension, hyperlipidemia, depression, Meniere’s disease, and insomnia.

## Discussion

The incidence rate for HL was higher in patients with FM, which confirms the association between FM and HL. This finding is consistent with that of previous studies and supports the hypothesis that patients with FM are at a higher risk of HL than those without FM [10–12]. Wolfe et al. reported that patients with FM had more sensory symptoms than those with rheumatoid arthritis or osteoarthritis [11]. However, in their study, HL was self-reported by the patients as “hearing difficulties within the last 6 months”; HL was not diagnosed by the physician. Stranden et al. reported that patients with FM showed an increase in subjective HL than those without FM or other musculoskeletal pain disorders [10]. The authors did not adjust for FM measured correctly by the physician, but adjusted for anxiety and depression symptoms, which were self-reported through questionnaires. Gencer et al. demonstrated that the patients with FM had significantly lower air conduction threshold values than the patients without FM at high frequencies [12], but they too did not adjust for other comorbidities and drugs.

The current result showed that sensorineural HL occurred commonly in patients with FM. This finding agrees with the results of Gerster & Hadl-Djilani [23] and Dohrenbusch et al. [24], who reported sensorineural HL in patients with FM in both high and low frequencies. Patients with FM may have sensorineural HL and auditory brainstem response abnormalities because of inner ear or central auditory damage [6]. This relationship may be partly explained by the notion that FM pathomechanism is related to central sensitization, including CNS sensory functions such as hearing. In addition, patients with FM in our cohort had conductive HL. Conductive HL is caused by problems in the outer ear, ear canal, or middle ear structures. Consequently, HL may have been caused by mechanisms involved in the pathogenesis of otoneurologic and systemic signs/symptoms of FM, which may induce abnormal perception of the stimuli from internal or external circumstances. Few patients in our cohort developed neural HL and central HL. Patients with HL had degenerative changes earlier in the central than in the peripheral auditory system [25]. Some studies have stated that patients with FM can report hearing-related complaints but display no objective defect in the audiometry [6, 13]. Similarly, measurement of auditory brainstem responses do not reveal changes in all patients with FM with hearing complaints. This makes it difficult to distinguish between peripheral and central origins in FM patients with hearing complaints.

Women and men with FM had a similar risk of HL compared with the non-FM group, consistent with the finding of Stranden et al. [10]. However, there is a considerably higher number of women with FM than men with FM [3], indicating higher numbers of women with FM who are at risk of HL than men with FM. Accordingly, clinicians should pay attention to the development of HL in women with FM.

In this study, the risk of HL was slightly greater in patients with FM aged ≤40 years than other age groups. By contrast, Stranden et al. demonstrated that the risk of HL increases with age in patients with FM [10]. A possible explanation for this discrepancy might be the high number of younger people in the current study with fewer HL-related comorbidities than their older counterparts, leading to a more pronounced effect of FM on the younger age group.

Some studies have reported results that corroborate the findings with HL-inducing drugs [21, 26, 27]. Our results revealed that patients taking antidepressants and HL-inducing drugs, such as anti-infectives, cancer drugs, loop diuretics, and aspirin, had a clear risk of HL. Moreover, patients taking pain-relieving drugs such as dopamine-receptor agonists, serotonin 5-HT3 receptor antagonists, antiepileptics, and opioid analgesics in this study had higher risks of HL, which was proposed by Oroei et al. and Hamed [28, 29]. The correlation between these drugs and HL development in FM needs further investigation.

FM has been considered a risk factor for HL [10–12], but no study has evaluated the combined effect of comorbidities in patients with FM on HL. The current study fills this gap and reveals that diabetes, hypertension, hyperlipidemia, depression, and Meniere’s disease increase the risk of HL in patients with FM both independently and jointly with FM. These conditions are often observed in combination with HL [16–19, 30], which is also commonly found in patients with FM [4, 31–33]. Moreover, HL is one of the most prevalent chronic health conditions in older adults [34], and the co-occurrence of two or more chronic conditions is common in older people [35]. The onset of FM is usually in the middle age [3]. Taken together, these data suggest that comorbidities and FM both contribute to HL development, and the joint effect in middle-aged or older adults might worsen the HL.

### Strengths and limitations

This study has some strengths. First, the FM and non-FM groups were matched for age, sex, and index year, which excludes the effects of these potential confounding variables. Second, the large sample size consolidates power for subgroup stratifications for further statistical analyses, which enabled us to determine the independent and joint effect of FM and comorbidities on HL development. Third, our study minimized the number of patients lost to follow-up because of the high coverage rate of the NHI program throughout the study.

However, this study had a few limitations. We could not control for other behavioral factors of HL risk, such as alcohol drinking, smoking, obesity, and physical activity, which were not obtained from the NHI dataset. Patients with FM and HL were enrolled based on physician diagnosis rather than through research assignment. Other factors not available in the NHIRD such as the stage of FM and neurotransmitter dysfunction could also have influenced the results.

Further research is required to validate the role of HL-related comorbidities/drugs in patients with FM in HL development. Additionally, control of other characteristics related to behavioral factors of HL, such as alcohol drinking, smoking, obesity, and physical activity, must be considered, which may affect the risk of HL in patients with FM. Furthermore, we recommend assessing all disease stages of FM and screening for neurotransmitter dysfunction to elucidate the relationship between HL and FM.

## Conclusion

The study results evidence that FM and a combination of FM and comorbidities presented a higher risk of HL. Additionally, FM was also found to have a higher incidence of sensorineural HL than other types of HL. These findings contribute to the understanding of the risk of HL in patients with FM and comorbidities and accordingly have several clinical implications. Health care professionals should provide appropriate screening for the risk of HL and prevention and counseling methods for patients with FM.

## Supporting information

S1 Appendix Joint effect of comorbidities on hearing loss. (DOCX)
