# Supplementary material for: Risk of hearing loss in patients with fibromyalgia: A nationwide population-based retrospective cohort study
Source: PLoS One. 2020 Sep 3;15(9):e0238502. doi: 10.1371/journal.pone.0238502 (PMC7470261; doi:10.1371/journal.pone.0238502)
Supplement: S1 Appendix — (DOCX) [file pone.0238502.s001.docx]

**S1 Appendix. Joint effect for hearing loss with comorbidity**

| Fibromyalgia | Diabetes | Hypertension | Hyperlipidemia | Depression | Meniere’s disease | Insomnia | N | Hearing loss no. | Rate | Adjusted HR (95% CI) | p | |
| --- | --- | --- | --- | --- | --- | --- | --- | --- | --- | --- | --- | --- |
| No | No | No | No | No | No | No | 77576 | 1370 | 1.94 | Ref. |  | |
| Yes | No | No | No | No | No | No | 31985 | 1177 | 3.07 | 1.63 (1.49-1.78) | <0.0001 | |
| No | Yes | No | No | No | No | No | 1753 | 54 | 4.16 | 1.26 (0.93-1.72) | 0.14 | |
| No | No | Yes | No | No | No | No | 10002 | 379 | 5.12 | 1.26 (1.10-1.44) | 0.0006 | |
| No | No | No | Yes | No | No | No | 2979 | 97 | 4.37 | 1.52 (1.20-1.93) | 0.0005 | |
| No | No | No | No | Yes | No | No | 635 | 19 | 4.10 | 1.60 (0.94-2.72) | 0.08 | |
| No | No | No | No | No | Yes | No | 1136 | 48 | 6.07 | 2.79 (2.08-3.74) | <0.0001 | |
| No | No | No | No | No | No | Yes | 1027 | 28 | 3.98 | 1.75 (1.16-2.62) | 0.007 | |
| Yes | Yes | No | No | No | No | No | 933 | 61 | 5.85 | 1.88 (1.40-2.51) | <0.0001 | |
| Yes | No | Yes | No | No | No | No | 5375 | 391 | 6.65 | 1.74 (1.53-1.99) | <0.0001 | |
| Yes | No | No | Yes | No | No | No | 2345 | 158 | 5.68 | 2.19 (1.82-2.63) | <0.0001 | |
| Yes | No | No | No | Yes | No | No | 644 | 26 | 3.41 | 1.73 (1.14-2.63) | 0.01 | |
| Yes | No | No | No | No | Yes | No | 1125 | 125 | 9.70 | 4.01 (3.27-4.94) | <0.0001 | |
| Yes | No | No | No | No | No | Yes | 1120 | 61 | 4.69 | 1.87 (1.39-2.53) | <0.0001 | |
| No | Yes | Yes | No | No | No | No | 2403 | 115 | 7.41 | 1.60 (1.28-1.98) | <0.0001 | |
| No | Yes | No | Yes | No | No | No | 1232 | 52 | 5.75 | 1.88 (1.38-2.56) | <0.0001 | |
| No | Yes | No | No | Yes | No | No | 40 | 1 | 3.62 | 1.42 (0.20-10.1) | 0.73 | |
| No | Yes | No | No | No | Yes | No | 85 | 8 | 16.01 | 3.71 (1.54-8.93) | 0.004 | |
| No | Yes | No | No | No | No | Yes | 77 | 5 | 11.59 | 4.68 (1.94-11.3) | 0.0006 | |
| No | No | Yes | Yes | No | No | No | 3204 | 156 | 6.68 | 1.77 (1.47-2.14) | <0.0001 | |
| No | No | Yes | No | Yes | No | No | 257 | 9 | 5.17 | 1.24 (0.61-2.49) | 0.55 | |
| No | No | Yes | No | No | Yes | No | 729 | 50 | 10.75 | 2.53 (1.86-3.44) | <0.0001 | |
| No | No | Yes | No | No | No | Yes | 595 | 29 | 7.70 | 1.85 (1.23-2.78) | 0.003 | |
| No | No | No | Yes | Yes | No | No | 71 | 2 | 4.24 | 1.85 (0.46-7.41) | 0.39 | |
| No | No | No | Yes | No | Yes | No | 145 | 10 | 11.38 | 3.71 (1.92-7.15) | <0.0001 | |
| No | No | No | Yes | No | No | Yes | 135 | 5 | 6.23 | 2.51 (1.04-6.05) | 0.04 | |
| No | No | No | No | Yes | Yes | No | 64 | 2 | 4.59 | 1.99 (0.50-7.97) | 0.33 | |
| No | No | No | No | Yes | No | Yes | 134 | 0 | 0.00 | NA |  | |
| No | No | No | No | No | Yes | Yes | 86 | 5 | 9.21 | 3.65 (1.52-8.79) | 0.004 | |
| Yes | Yes | Yes | No | No | No | No | 1147 | 115 | 10.15 | 2.30 (1.86-2.86) | <0.0001 | |
| Yes | Yes | No | Yes | No | No | No | 780 | 60 | 6.60 | 2.46 (1.87-3.25) | <0.0001 | |
| Yes | Yes | No | No | Yes | No | No | 23 | 2 | 7.58 | 3.04 (0.76-12.2) | 0.12 | |
| Yes | Yes | No | No | No | Yes | No | 62 | 10 | 15.43 | 5.84 (3.13-10.9) | <0.0001 | |
| Yes | Yes | No | No | No | No | Yes | 81 | 9 | 10.64 | 2.19 (0.91-5.29) | 0.08 | |
| Yes | No | Yes | Yes | No | No | No | 2255 | 200 | 7.88 | 2.10 (1.78-2.49) | <0.0001 | |
| Yes | No | Yes | No | Yes | No | No | 165 | 12 | 6.75 | 2.02 (1.14-3.59) | 0.02 | |
| Yes | No | Yes | No | No | Yes | No | 625 | 79 | 12.53 | 2.94 (2.29-3.77) | <0.0001 | |
| Yes | No | Yes | No | No | No | Yes | 554 | 56 | 10.00 | 2.32 (1.72-3.13) | <0.0001 | |
| Yes | No | No | Yes | Yes | No | No | 80 | 11 | 12.13 | 4.23 (2.19-8.18) | <0.0001 | |
| Yes | No | No | Yes | No | Yes | No | 217 | 28 | 11.38 | 4.62 (3.15-6.78) | <0.0001 | |
| Yes | No | No | Yes | No | No | Yes | 183 | 15 | 6.95 | 1.99 (1.03-3.84) | 0.04 | |
| Yes | No | No | No | Yes | Yes | No | 82 | 11 | 11.87 | 5.91 (3.33-10.5) | <0.0001 | |
| Yes | No | No | No | Yes | No | Yes | 204 | 15 | 6.55 | 2.56 (1.44-4.54) | 0.001 | |
| Yes | No | No | No | No | Yes | Yes | 119 | 10 | 7.50 | 3.65 (2.01-6.62) | <0.0001 | |
| No | Yes | Yes | Yes | No | No | No | 2777 | 132 | 7.01 | 1.75 (1.43-2.14) | <0.0001 | |
| No | Yes | Yes | No | Yes | No | No | 80 | 5 | 11.28 | 2.24 (0.84-6.00) | 0.1 | |
| No | Yes | Yes | No | No | Yes | No | 232 | 13 | 9.37 | 2.50 (1.44-4.32) | 0.001 | |
| No | Yes | Yes | No | No | No | Yes | 174 | 6 | 5.79 | 1.21 (0.5-2.91) | 0.68 | |
| No | Yes | No | Yes | Yes | No | No | 43 | 2 | 7.40 | 1.58 (0.22-11.2) | 0.65 | |
| No | Yes | No | Yes | No | Yes | No | 83 | 3 | 6.00 | 2.12 (0.68-6.58) | 0.19 | |
| No | Yes | No | Yes | No | No | Yes | 69 | 8 | 18.32 | 4.53 (1.88-10.9) | 0.0008 | |
| No | Yes | No | No | Yes | Yes | No | 8 | 0 | 0.00 | NA |  | |
| No | Yes | No | No | Yes | No | Yes | 10 | 0 | 0.00 | NA |  | |
| No | Yes | No | No | No | Yes | Yes | 8 | 1 | 17.20 | 5.3 (0.75-37.64) | 0.1 | |
| No | No | Yes | Yes | Yes | No | No | 74 | 3 | 6.31 | 1.93 (0.62-6.01) | 0.26 | |
| No | No | Yes | Yes | No | Yes | No | 365 | 29 | 12.49 | 3.04 (2.03-4.57) | <0.0001 | |
| No | No | Yes | Yes | No | No | Yes | 279 | 18 | 9.69 | 3.07 (1.92-4.90) | <0.0001 | |
| No | No | Yes | No | Yes | Yes | No | 70 | 6 | 13.38 | 2.91 (1.20-7.01) | 0.02 | |
| No | No | Yes | No | Yes | No | Yes | 80 | 3 | 5.64 | 1.01 (0.25-4.05) | 0.99 | |
| No | No | Yes | No | No | Yes | Yes | 117 | 5 | 7.31 | 1.48 (0.55-3.95) | 0.44 | |
| No | No | No | Yes | Yes | Yes | No | 9 | 0 | 0.00 | NA |  | |
| No | No | No | Yes | Yes | No | Yes | 17 | 0 | 0.00 | NA |  | |
| No | No | No | Yes | No | Yes | Yes | 29 | 0 | 0.00 | NA |  | |
| No | No | No | No | Yes | Yes | Yes | 25 | 2 | 15.22 | 7.99 (1.99-32.0) | 0.003 | |
| Yes | Yes | Yes | Yes | No | No | No | 1747 | 159 | 8.55 | 2.05 (1.69-2.47) | <0.0001 | |
| Yes | Yes | Yes | No | Yes | No | No | 63 | 9 | 15.60 | 4.34 (2.24-8.40) | <0.0001 | |
| Yes | Yes | Yes | No | No | Yes | No | 191 | 22 | 12.44 | 2.81 (1.82-4.35) | <0.0001 | |
| Yes | Yes | Yes | No | No | No | Yes | 126 | 11 | 9.17 | 1.6 (0.80-3.22) | 0.19 | |
| Yes | Yes | No | Yes | Yes | No | No | 25 | 1 | 3.22 | 1.19 (0.17-8.46) | 0.86 | |
| Yes | Yes | No | Yes | No | Yes | No | 103 | 13 | 11.11 | 3.95 (2.23-6.98) | <0.0001 | |
| Yes | Yes | No | Yes | No | No | Yes | 82 | 6 | 6.38 | 2.50 (1.12-5.59) | 0.03 | |
| Yes | Yes | No | No | Yes | Yes | No | 3 | 0 | 0.00 | NA |  | |
| Yes | Yes | No | No | Yes | No | Yes | 15 | 2 | 12.65 | 1.84 (0.26-13.1) | 0.54 | |
| Yes | Yes | No | No | No | Yes | Yes | 14 | 0 | 0.00 | NA |  | |
| Yes | No | Yes | Yes | Yes | No | No | 86 | 9 | 9.39 | 2.35 (1.17-4.74) | 0.02 | |
| Yes | No | Yes | Yes | No | Yes | No | 406 | 49 | 11.34 | 2.78 (2.04-3.79) | <0.0001 | |
| Yes | No | Yes | Yes | No | No | Yes | 300 | 22 | 6.68 | 1.75 (1.11-2.76) | 0.02 | |
| Yes | No | Yes | No | Yes | Yes | No | 50 | 7 | 13.75 | 3.84 (1.82-8.11) | 0.0004 | |
| Yes | No | Yes | No | Yes | No | Yes | 88 | 8 | 8.76 | 2.35 (1.17-4.73) | 0.02 | |
| Yes | No | Yes | No | No | Yes | Yes | 162 | 21 | 13.07 | 3.34 (2.16-5.17) | <0.0001 | |
| Yes | No | No | Yes | Yes | Yes | No | 29 | 5 | 15.34 | 6.94 (2.87-16.8) | <0.0001 | |
| Yes | No | No | Yes | Yes | No | Yes | 31 | 2 | 5.84 | 2.56 (0.64-10.3) | 0.19 | |
| Yes | No | No | Yes | No | Yes | Yes | 27 | 5 | 16.85 | 5.01 (1.88-13.4) | 0.001 | |
| Yes | No | No | No | Yes | Yes | Yes | 48 | 10 | 19.92 | 7.45 (3.7-14.99) | <0.0001 | |
| No | Yes | Yes | Yes | Yes | No | No | 92 | 4 | 6.95 | 1.94 (0.73-5.19) | 0.19 | |
| No | Yes | Yes | Yes | No | Yes | No | 333 | 23 | 12.21 | 2.71 (1.72-4.28) | <0.0001 | |
| No | Yes | Yes | Yes | No | No | Yes | 249 | 11 | 8.14 | 2.12 (1.14-3.96) | 0.02 | |
| No | Yes | Yes | No | Yes | Yes | No | 13 | 1 | 12.41 | 4.88 (1.22-19.6) | 0.03 | |
| No | Yes | Yes | No | Yes | No | Yes | 26 | 0 | 0.00 | NA |  | |
| No | Yes | Yes | No | No | Yes | Yes | 33 | 0 | 0.00 | NA |  | |
| No | Yes | No | Yes | Yes | Yes | No | 10 | 0 | 0.00 | NA |  | |
| No | Yes | No | Yes | Yes | No | Yes | 11 | 0 | 0.00 | NA |  | |
| No | Yes | No | Yes | No | Yes | Yes | 8 | 1 | 22.38 | 10.1 (1.42-71.5) | 0.02 | |
| No | Yes | No | No | Yes | Yes | Yes | 5 | 0 | 0.00 | NA |  | |
| No | No | Yes | Yes | Yes | Yes | No | 30 | 2 | 12.03 | 3.45 (0.86-13.9) | 0.08 | |
| No | No | Yes | Yes | Yes | No | Yes | 50 | 1 | 3.20 | 1.05 (0.15-7.45) | 0.96 | |
| No | No | Yes | Yes | No | Yes | Yes | 76 | 3 | 7.35 | 1.37 (0.34-5.48) | 0.66 | |
| No | No | Yes | No | Yes | Yes | Yes | 38 | 3 | 15.39 | 3.41 (1.09-10.6) | 0.03 | |
| No | No | No | Yes | Yes | Yes | Yes | 4 | 0 | 0.00 | NA |  | |
| Yes | Yes | Yes | Yes | Yes | No | No | 90 | 13 | 14.35 | 4.10 (2.36-7.12) | <0.0001 | |
| Yes | Yes | Yes | Yes | No | Yes | No | 350 | 54 | 15.36 | 3.52 (2.61-4.75) | <0.0001 | |
| Yes | Yes | Yes | Yes | No | No | Yes | 234 | 15 | 6.01 | 1.43 (0.82-2.47) | 0.21 | |
| Yes | Yes | Yes | No | Yes | Yes | No | 17 | 0 | 0.00 | NA |  | |
| Yes | Yes | Yes | No | Yes | No | Yes | 23 | 4 | 19.63 | 4.08 (1.31-12.7) | 0.02 | |
| Yes | Yes | Yes | No | No | Yes | Yes | 52 | 14 | 32.11 | 6.71 (3.87-11.6) | <0.0001 | |
| Yes | Yes | No | Yes | Yes | Yes | No | 5 | 0 | 0.00 | NA |  | |
| Yes | Yes | No | Yes | Yes | No | Yes | 17 | 0 | 0.00 | NA |  | |
| Yes | Yes | No | Yes | No | Yes | Yes | 14 | 2 | 13.22 | 2.37 (0.33-16.8) | 0.39 | |
| Yes | Yes | No | No | Yes | Yes | Yes | 9 | 2 | 21.18 | 8.38 (2.09-33.6) | 0.003 | |
| Yes | No | Yes | Yes | Yes | Yes | No | 37 | 9 | 24.90 | 5.16 (2.56-10.4) | <0.0001 | |
| Yes | No | Yes | Yes | Yes | No | Yes | 68 | 6 | 8.08 | 2.29 (1.02-5.13) | 0.04 | |
| Yes | No | Yes | Yes | No | Yes | Yes | 102 | 10 | 9.24 | 2.38 (1.27-4.44) | 0.007 | |
| Yes | No | Yes | No | Yes | Yes | Yes | 35 | 5 | 13.50 | 2.52 (0.94-6.77) | 0.07 | |
| Yes | No | No | Yes | Yes | Yes | Yes | 7 | 2 | 29.29 | 11.0 (2.75-44.2) | 0.0007 | |
| No | Yes | Yes | Yes | Yes | Yes | No | 32 | 2 | 12.02 | 1.30 (0.18-9.27) | 0.79 | |
| No | Yes | Yes | Yes | Yes | No | Yes | 38 | 3 | 14.95 | 2.73 (0.68-11.0) | 0.16 | |
| No | Yes | Yes | Yes | No | Yes | Yes | 63 | 4 | 12.60 | 0.81 (0.11-5.76) | 0.83 | |
| No | Yes | Yes | No | Yes | Yes | Yes | 8 | 0 | 0.00 | NA |  | |
| No | Yes | No | Yes | Yes | Yes | Yes | 1 | 0 | 0.00 | NA |  | |
| No | No | Yes | Yes | Yes | Yes | Yes | 24 | 1 | 6.93 | 1.89 (0.27-13.4) | 0.53 | |
| Yes | Yes | Yes | Yes | Yes | Yes | No | 46 | 5 | 10.37 | 1.56 (0.50-4.84) | 0.45 | |
| Yes | Yes | Yes | Yes | Yes | No | Yes | 50 | 4 | 8.38 | 1.62 (0.52-5.03) | 0.41 | |
| Yes | Yes | Yes | Yes | No | Yes | Yes | 96 | 19 | 19.95 | 4.48 (2.76-7.26) | <0.0001 | |
| Yes | Yes | Yes | No | Yes | Yes | Yes | 11 | 2 | 21.47 | 2.12 (0.3-15.06) | 0.45 | |
| Yes | Yes | No | Yes | Yes | Yes | Yes | 3 | 2 | 93.69 | 29.29 (7.30-117) | <0.0001 | |
| Yes | No | Yes | Yes | Yes | Yes | Yes | 38 | 10 | 26.84 | 6.50 (3.36-12.6) | <0.0001 | |
| No | Yes | Yes | Yes | Yes | Yes | Yes | 18 | 2 | 27.20 | 7.45 (1.86-29.9) | 0.005 | |
| Yes | Yes | Yes | Yes | Yes | Yes | Yes | 34 | 3 | 9.41 | 2.12 (0.68-6.60) | 0.2 | |
| Adjusted for age, gender, anti-depression drug, pain-releasing drug, and hearing loss-inducing drug | | | | | | | | | | | |  |
